# Indefinite Pain, Its Cause as Viewed by the Dowd Phosphatometer*Read at 8th Dist. Meeting N. Y. State Medical Society, Olean, N. Y.

**Published:** 1916-10

**Authors:** B. E. Smith

**Affiliations:** Surgeon N. Y. C. Lines; Health Commissioner, Angola, N. Y.


### Indefinite Pain, Its Cause as Viewed by the Dowd Phosphatometer.*

DR. B. E. SMITH,

Surgeon N. Y. C. Lines; Health Commissioner,

Angola, N. Y.

Pain is defined as a local sensation of distress due to injury or disease; the expression of an abnormally severe impression on a sensory nerve, exclusive of nausea, feeling of distress, itching, etc., although it may be associated with any of these. It is thus seen that nerves must be involved before pain is evidenced, and it is only where we find nerves that we find pain. Thus, it is possible to cut the hair, nails, cornia, and epithelium without pain, but in these we are not concerned.

Amplifying the cause of pain from that already stated, it is found to be due to injury, inflammation, pressure, disease, but above all, and the most important cause, is insufficient nourishment of the nerve centers. In other words, where nerves are hungry, as they cannot speak, they manifest their want of food by pain. This is easily and quickly proven in the condition known as neuritis where injury, pressure and disease can be eliminated; and which is possible in fully 90% of all cases; they quickly respond to nerve nourishment. The question now arises, how can we say the nerve cells are at fault, hungry, so to speak, and that the pain is due to the nerves calling for food?

It is true we are taught the nerve cells have a food; we know this food is lecithin, nuclein and phosphorus, but until the original articles of Dr. Dowd (N. Y. Med. Record 1908) nothing was known of any way of ascertaining whether these substances were present in normal quantities or not. One thing his teachings have brought clear to us; these substances are taken from the food we eat, not only in sufficient quantities to maintain the daily calls for energy, but an excess is taken which is stored for emergencies. That such a condition had been supposed, is evident from the writings of Prof. Shaw in his work, when speaking of neurasthenia, a condition that owes its cause in fully 90% of cases to nerve cell hunger. He says, "This inherited unstable state of the nervous system depends evidently upon some defect in the power of the neurones to assimilate and store up nutrition and force in sufficient quantity, and with sufficient rapidity to carry on fully and easily the work of life."

*Read at 8th Dist. Meeting N. Y. State Medical Society, Olean, N. Y.

As to whether nerve nourishment is present and being used by the system in normal amounts, we find easily and quickly ascertainable by estimation of the alkaline phosphates found in the urine. These represent the end products of lecithin, nuclein and phosphorus, occurring through a process of metabolism and will show the condition of the nervous system in exactly the same manner as does urea show muscular activity.

Of course, in pain accompanying injury, inflammation, irritation and the like, the cause is evident and need not be considered here, except in cases where it is indefinitely prolonged; here it may be found the phosphatometer may give most valuable information, and clearly show what to do for its relief. But it is the great majority of conditions where pain is present, and where no apparent cause is evident—fully 70%—that we can look to the nervous system as being at fault.

Barring neuritis and neuralgia, rheumatism is the cause given in most cases of indefinite pain. This is a sad error, and there is no doubt of the statement made some time ago by a prominent writer, ''Eight cases out of ten treated as rheumatism, are not rheumatism at all, but nerve involvement; the majority are due to nerve-cell impoverishment, and the pain is due to the nerve crying for food.''

Practically all pain of an indefinite nature being charged to rheumatism, and due to that old exploded bugaboo, uric acid excess, let me confine my further remarks to the pseudo-rheumatic opinion.

When physicians from small places, commonly called the country doctor, visits the cities and hears reputed authorities make remarks as heretofore noted, he thinks, thinks long and deeply; possibly going over in his mind, Mrs. So-and-So's trouble must have been of a nervous character, rather than rheumatism for which I treated her, otherwise she would have recovered in my hands, instead of Dr. Blank's. It was a thought of this kind I had, for many cases of pains and aches, which were diagnosticated as rheumatism had not responded as I thought they should.

It was some time before this happening that I had seen an article ''The Neuroses,'' by Dr. Talbot, New York State Journal of Medicine, which among other things said, ''Pain is always due to nerve involvement, and when there is not a well defined cause, as an injury, inflammation and the like, in the great majority of cases it is due to the nerves crying for food.''

One thing can be stated as a fact: If digestion and assimilation are normal, the calls for energy not excessive, nerve nutrition will be furnished from the food we consume, and in sufficient quantities to maintain all functions normally.

All physicians realize that the nerves cannot speak, neither can the baby; when it is irritated or hungry, it cried; when the former, they also cry, cry in the form of pains and aches.

One thing we are positively sure of, true rheumatism responds quicly to anti-rheumatic remedies; therefore, in any case of indefinite or prolonged pain, or pain that does not respond to rational medication within a reasonable time, it is well to think of the nervous system. But the day of thinking in medicine is fast drawing to a close, and conditions of the nervous system, especially that great class described as functional, accompanied by pains and aches, heretofore treated as rheumatism, will quickly be relieved when the true cause is ascertained.

If I am not mistaken, it was some seven or eight years ago that Dr. J. Henry Dowd, of Buffalo, gave to the medical profession what is known as the Phosphatic Index, or a test for the alkaline phosphates found in the urine, and which show the condition of the nervous system, whether nerve nutrition is present in normal quantities and is being used in the same manner.

Although the subject is free from many, if not all the technicalities that go with most tests we are given today; so simple that a medical student can use it, I feel that I have not the time or space, and these are among Dr. Dowd's remarks: "The nervous system has a fad which must be supplied in sufficient quantities at all times to furnish the necessary energy to keep this system, which furnishes every organ, controls every function of the human body, in as normal a condition as it is to have it." It is very evident that if nerve energy is used faster than it is supplied but one condition results: sooner or later there is a starvation process present and the nerves will make their wants known, possibly an irritability to care for the food consumed—dyspepsia—possibly by a general weakness, "constantly tired," but usually by pain; indefinite pains in the back, lumbago; in localized nerves, as neuritis, neuralgia and the like, and at times involving all parts of the body, erroneously diagnosed and treated as rheumatism.

The estimation of the alkaline phosphates is a simple matter by use of the Phosphatometer, a graduated tube showing normal, plus and minus; excessive destruction of nerve nutrition or a want of it.

And here it may be noted, as the nervous system is the most important part of our make-up, the vital spark of life—and a system that is influenced solely by impressions from any and every thought, word or deed; in the great majority of cases, some authorities say at least 80%, it will be found the nerve cells are impoverished, that is, they are below par in nourishment.

It is unnecessary to describe the modus operandi for taking the Phosphatic Index, the subject is well known and most explicit instructions accompany each apparatus.

A word as to nerve food when it must be given artificially. Lecithin and nuclein are best obtained from raw eggs. As to phosphorus, of late the impression is fast gaining ground that this is one of the most valuable drugs we have, and time will prove such to be a fact; but when it is used, it must be used as such and not as a compound, if results are to be forthcoming. We, as physicians, have been deluded into thinking that we have obtained phosphorus from such compounds as hypo-glycerophosphates, phosphoric acid mixtures, and the like. Happily to say, with new light shed upon this subject through editorials and otherwise, it is clear to us that the above contain very little, if any, elementary phosphorus. This is easily proven with the mixture in the case report. If dropped in water it emits a bluish smoke, characteristic fumes and becomes decidedly luminous in the dark. (What a pleasure it would be if the physician could prove other mixtures contained what they purported to.)

The following case reports show with what rapidity results are obtained when the true cause is ascertained:

This lady had been ailing for nearly a year; four or five physicians having treated her condition as rheumatism; six weeks had been spent in a sanitarium for this malady, but no result. She was becoming worse every day, until the condition was most pitiable. All small joints were enlarged and very painful at times; the elbows were involved to such an extent that dressing herself was impossible without aid; she could not comb her hair, could scarcely attend to household duties; it was almost impossible to walk so badly were her feet swollen. She was very anemic, no appetite and obstinate constipation. Careful examination showed no pathological condition present; she was hysterical, crying several times daily. Careful examination of the urine showed no excess of uric acid, no evidence of Bright's, in fact nothing of an inflammatory nature was found. The index was 90% minus Sp. G. 1018, urine alkaline. This showed what is known as nerve-cell starvation, nerve nourishment was 90% below normal, pain was due to the nerves crying for food.

The following was ordered taken three times daily, about half an hour after meals: Comp. Phos. Tonic (Dowd) M 25, Fl. Ex. Valerian M. 15, Res. Podoph, gr. 1-10 in glycerine to one drachm, also two raw eggs a day. It will be unnecessary to go into detail further than to say: she commenced to improve at once, at the end of a week the pain had vanished, in two weeks the swelling had entirely disappeared from the joints and she had gained 20 pounds and felt perfectly well.

A rather amusing, but welcome condition followed. She had been married three years, but never pregnant. Three months after commencing the phosphorus mixture, she reported a miss in her menstrual function. Examination showed pregnancy, all the crystals being stripped of their feathery ends; about eight months afterwards a boy was born. Is it not possible the ovaries were in a dormant condition, due to the lowered condition of the vital forces; improvement in her condition produced ovulation of an animated nature.

Another lady (aet 55) with almost similar symptoms, only not quite so severe, accompanied by dizziness, flushing of the face from which she had suffered for over a year, had been treated for rheumatism, neuritis and what not, with no result; she had even undergone the starvation method for high blood pressure; this registered 190 constantly. Examination showed interstitial nephritis with an index of 175% plus, solid. This case showing nerve cell irritation, excessive metabolism, bromide of gold and arsenic was used. In three weeks she was quite free of pain and dizziness; blood pressure was reduced to 160, she felt fairly good, living for two years in comparative comfort, when she suddenly died of apoplexy. It might be noted she only took the gold mixture for about five weeks.

THE FOLLOWING CONCLUSIONS MAY BE BRIEFLY STATED:

A. Fully 70% of indefinite pain is due to nerve cell involvement, irritation or want of nutrition; the Phosphatometer will quickly decide the true cause.

B. In fully 60% of these conditions it is due to a want of nutrition; the administration of nerve-cell food will be followed by rapid and permanent results.

C. About 10% are due to nerve-cell irritation and calls for sedatives; if condition is acute, bromides or valerian; if chronic, bromide of gold and arsenic.

D. In high blood pressure the Index is always plus, solid above N. P.; the Phosphatometer shows positively what treatment should be given. The happiest of results follow rational medication, in contradistinction to the heretofore starvation method used for this condition.

E. The Phosphatometer has brought to the door of the general practitioner the most simple, yet scientific method for the relief of obscure conditions of pain.

F. The writer can fully endorse the opinion of Dr. Chauncey Pelton Smith, University of Pennsylvania, ''Taking the phosphatic index should be a routine of all medical examinations—it gives more information, in obscure conditions, than any other measure we have.''

Angola, N. Y.

---

**Horse Chestnuts as Food.** H. Serger, Chem. Zeit. gives the analysis as Starch 42%, saccharine matter 9%, proteid 5%, oil 2.5%, mineral matter 1.5%, water 40%. There are also a bitter principle and saponin-like glucosides. The cracked nuts, soaked in water, may be sufficiently freed from the latter to be available for live stock and extraction with 50% alcohol which, of course can be redistilled, allows the preparation of a flour making good bread for human beings. A tree 100 ft. high, yields about 3000-4000 nuts, weighing 40 kilos and yielding about 10 kilos of flour. While in peace, the labor of collecting and preparing the nuts renders the expense too great, in war, the horse chestnut may be worth utilizing as food, at least for live stock. (Note: So far as our observation goes, the horse chestnut is much more common in western Europe than in America. There is prepared a paste, used as a dessert, having the characteristic flavor and bearing on the packages a picture of a horse chestnut spray).

---

**Absence of Ossification in an Adult.** Pl. Mauclaire, Soc. Chir., Dec. 22, 1915, describes the case of a soldier under observation for a shrapnel wound of the perineum. The protrusion of both tibial tuberosities attracted attention and X-ray examination showed the osseous centers of the tuberosities distinctly separate from the shafts. The patient complained only of occasional pains in the knee.

Traumatic Luxation of Penis. Pl. Maucaire, Soc. Chir., Jan. 5, 1916, describes the case of a soldier who received, at the same time, a ball in the penis and one in the left testicle and a cut in the same region. Enormous tumefaction ensued, and the penis had come out of its sheath, while the left testicle was in process of enucleation. The sheath was cut at its base, on the lower portion of its circumference and the whole penis enucleated. An autoplastic operation will be performed later.

---

**Abscess of Liver—''Nostras.''** Muller, ibid., reports a case in a man of 27, who had not been out of France. A large abscess of the left lobe was drained and recovery ensued. The origin is ascribed to an attack of appendicitis, with removal, 3 years previously.

